# Laparoscopic round‑ligament duodenostomy synchronized with posterior mediastinal reconstruction

**DOI:** 10.20452/wiitm.2025.17999

**Published:** 2025-12-16

**Authors:** Takeshi Matsubara, Yoko Senaha, Hiroki Okamura, Shunsuke Kaji, Hikota Hayashi, Kazunari Ishitobi, Takahito Taniura, Takayuki Tanaka, Tetsu Yamamoto, Masaaki Hidaka

**Affiliations:** Department of Digestive and General Surgery, Faculty of Medicine Shimane University Japan

**Keywords:** duodenostomy, enteral nutrition, esophagectomy, minimally‑invasive surgery, round ligament

## Abstract

**INTRODUCTION:**

Esophagectomy is a highly invasive procedure, and early enteral nutrition supports recovery. Feeding jejunostomy is common but may cause fixation-related mechanical complications. To address these issues, a duodenostomy using the hepatic round ligament was adapted to a fully laparoscopic approach.

**AIM:**

We aimed to evaluate the feasibility and short-term outcomes of laparoscopic duodenostomy using the round ligament as enteral access during esophagectomy.

**MATERIALS AND METHODS:**

We retrospectively reviewed 26 consecutive patients who underwent esophagectomy with duodenostomy at a single institution: 15 by a standardized laparoscopic technique and 11 by historical minilaparotomy. The laparoscopic method routes a catheter through a round-ligament sleeve, with double purse-string fixation at the duodenal bulb and 3-point anchoring at the intestinal and abdominal wall sites. The primary outcomes were feasibility and timing of enteral feeding initiation. A tube-related infection was defined as local redness, swelling, purulent discharge, or abscess along the catheter tract or exit site, consistent with the Centers for Disease Control and Prevention criteria. Noninfectious tube-related complications included dislodgement, inversion, or obstruction due to kinking. Differences between the groups are presented descriptively.

**RESULTS:**

All laparoscopic procedures achieved successful catheter placement. Enteral feeding began earlier after laparoscopy (median [interquartile range] postoperative day, 1 [1–2]) than minilaparotomy (2 [2–6]). Tube-related infection occurred in 0 of 15 laparoscopy procedures and 2 of 11 (18.2%) minilaparotomies, and noninfectious tube-related complications occurred in 1 of 15 patients (6.7%) from the former group and 1 of 11 (9.1%) from the latter.

**CONCLUSIONS:**

In this small, single-center, retrospective, exploratory series, laparoscopic round-ligament duodenostomy was feasible and coherent with minimally-invasive esophagectomy, and may facilitate earlier enteral access while reducing fixation-related problems; these findings require confirmation in larger prospective studies.

## INTRODUCTION 

Esophagectomy for esophageal cancer is highly invasive and commonly involves extensive lymph node dissection. Despite advances in minimally-invasive techniques and perioperative care, postoperative complications remain prevalent and are often exacerbated by malnutrition and immunosuppression related to dysphagia and anorexia.^1^^,^^2^^,^^3^^,^^4^^,^^5^

Enteral nutrition-most commonly via feeding jejunostomy-can enhance recovery and reduce complications such as pneumonia and anastomotic leakage.^6^ However, jejunostomy carries risks including bowel obstruction from kinking, adhesions, or volvulus, with reported rates of 3.7% − 11.5% that may necessitate reoperation.^7^^,^^8^^,^^9^^,^^10^^,^^11^^,^^12^

**FIGURE 1:**
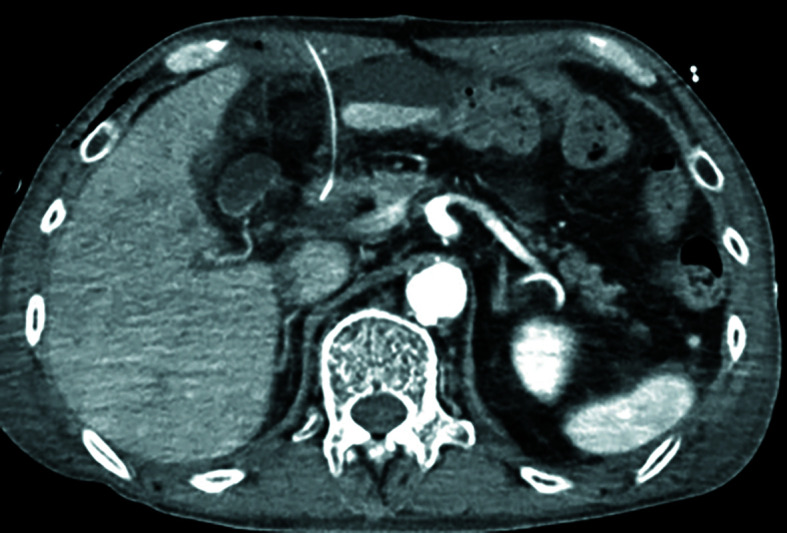
Abscess formation along the enterostomy tract (arrow)

Until 2021, jejunostomy had been our default route for enteral access after esophagectomy, but we encountered device-related problems, such as mechanical small bowel obstruction related to abdominal wall fixation, exit site infection, and occasional dislodgement. To mitigate these issues, we introduced duodenostomy using the hepatic round ligament via minilaparotomy in 2021; however, 2 patients developed surgical site infection FIGURE 1. We subsequently adopted a fully laparoscopic duodenostomy using the round ligament to improve visualization, reduce surgical stress, and avoid an upper-abdominal incision. While round ligament duodenostomy has been described via minilaparotomy, the laparoscopic variant may provide magnified visualization and incision sparing advantages. Since 2022, laparoscopic round-ligament duodenostomy has been our default approach. Although jejunostomy was not analyzed as a comparative procedure in this study, our transition was motivated by recurring fixation-related problems with jejunostomy.

**FIGURE 2:**
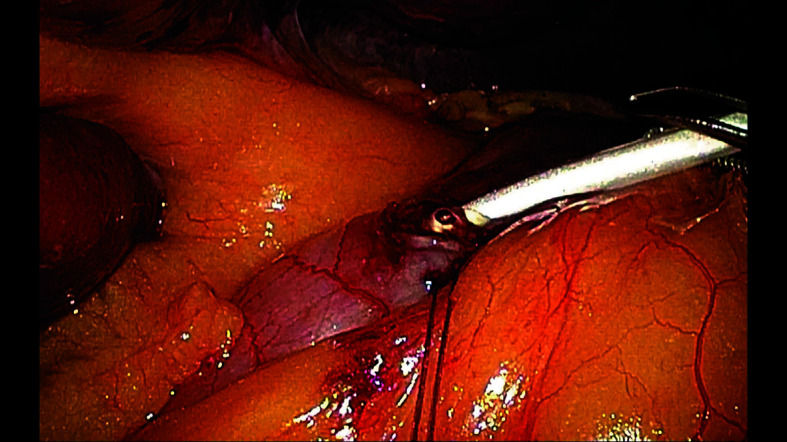
A purse-string suture placed at the duodenal bulb, and the catheter advanced 20 − 30 cm beyond the ligament of Treitz into the jejunum

**FIGURE 3:**
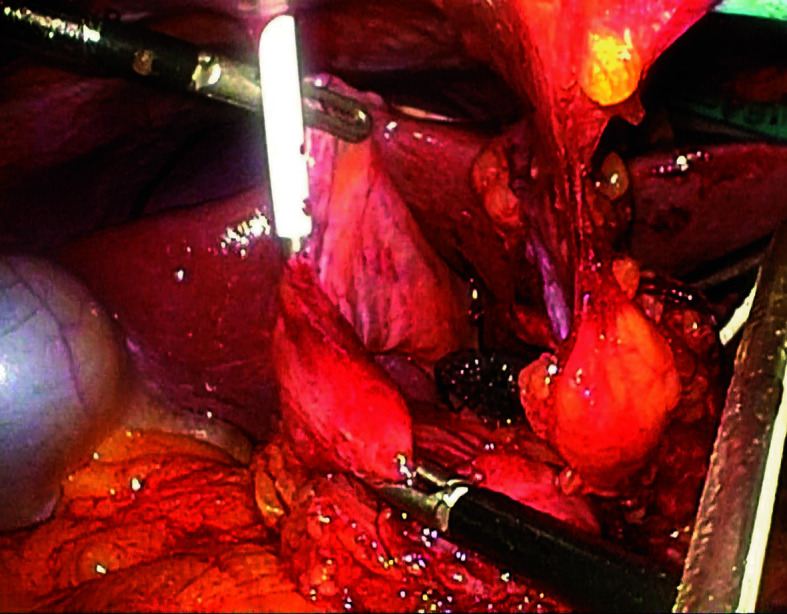
Dissected round ligament (arrow) punctured percutaneously with a plastic cannula under laparoscopic guidance to create a pathway for the catheter

## AIM 

We aimed to evaluate the feasibility and short-term outcomes of laparoscopic duodenostomy using the hepatic round ligament as enteral access during esophagectomy, and to describe a standardized technique, with descriptive comparison with an early minilaparotomy series.

## MATERIALS AND METHODS 

### Study design and patients 

We conducted a retrospective review of 26 consecutive patients who underwent esophagectomy with round-ligament duodenostomy at a single center between 2021 and 2025, using either minilaparotomy ( n = 11 ) or a fully laparoscopic technique ( n = 15 ). Patient demographics, including age, sex, and American Society of Anesthesiologists physical status (ASA-PS), as well as perioperative outcomes and complications were obtained from the medical records. The study adhered to the Declaration of Helsinki, and was approved by the Ethics Review Board of the Shimane University Faculty of Medicine (20231226-1); the need for individual consent was waived with an opt-out notice according to local regulations.

Outcomes and definitions The primary feasibility end point was successful catheter placement. Early enteral access was assessed as the postoperative day (POD) of initiation of continuous feeding. A tube-related infection was defined as local redness, swelling, purulent discharge, or abscess along the catheter tract or exit site, consistent with the Centers for Disease Control and Prevention surgical site infection criteria. Noninfectious tube-related complications included dislodgement, inversion, or obstruction due to kinking.

**FIGURE 4:**
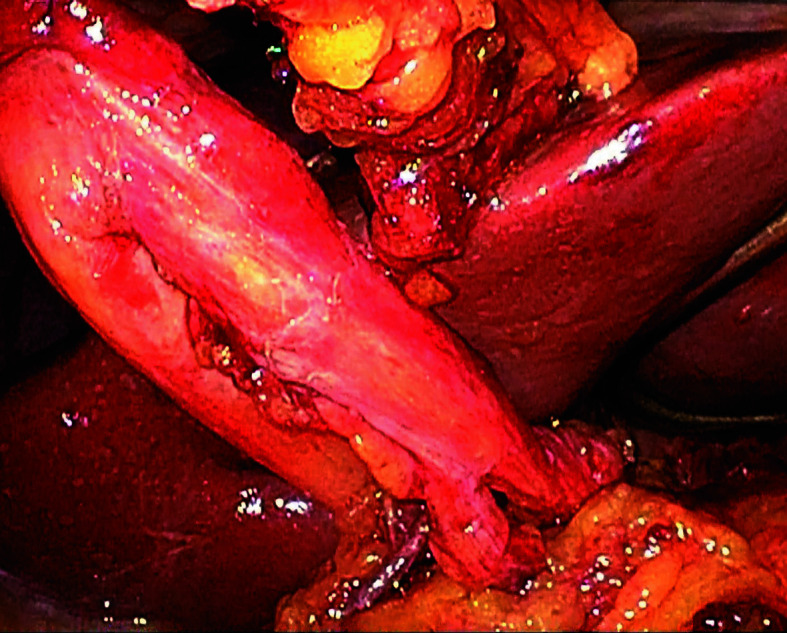
The enterostomy tube covered by the hepatic round ligament and secured on both the abdominal wall and duodenal sides (arrows)

**FIGURE 5:**
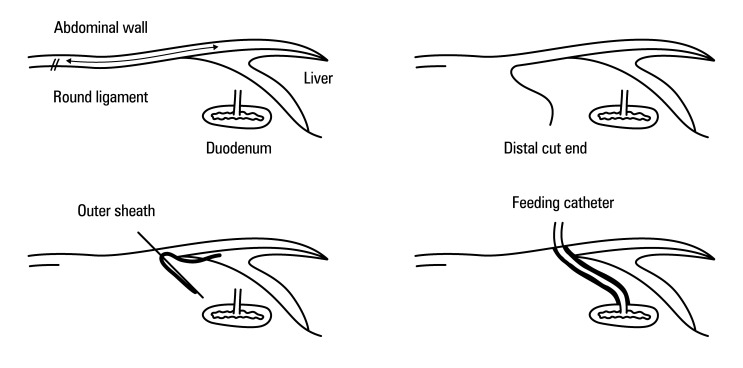
Schematic illustration of laparoscopic round-ligament duodenostomy; A - a feeding catheter (blue arrow) is first inserted into the duodenum through purse-string duodenostomy; the black arrow denotes the dissection line and // symbol denotes the cut line of the round ligament; B, C - the hepatic round ligament (red arrow) is then mobilized from the abdominal wall and divided at its distal end to create a tunnel between the abdominal wall and the duodenostomy site. An outer sheath advanced through this tunnel (yellow arrow) is used to exteriorize the feeding catheter; D - finally, the catheter is covered and anchored by the hepatic round ligament, which forms a sleeve between the abdominal wall and the duodenum (arrow).

Fully laparoscopic technique After constructing a narrow gastric conduit, it was elevated to the neck through the posterior mediastinal route. The hepatic round ligament was laparoscopically mobilized and transected near the umbilicus. A 9Fr silicone catheter (eg, Kangaroo jejunostomy catheter; Covidien, Mansfield, Massachusetts, United States) was used. A purse-string suture was placed on the duodenal bulb, and the catheter was advanced 20 − 30 cm beyond the ligament of Treitz into the jejunum. Correct intraluminal positioning of the catheter tip was confirmed by gentle saline injection under laparoscopic assistance, ensuring free intraluminal flow without resistance or leakage around the duodenal bulb; in selected cases, a guidewire was used to facilitate smooth advancement when resistance was encountered. The second purse-string suture secured the catheter at the bulb. Under laparoscopic assistance, the round ligament was punctured percutaneously with a plastic cannula to create a round-ligament sleeve pathway. The catheter was routed through this sleeve. The ligament was anchored to the duodenum around the catheter with 3 seromuscular fixation sutures (absorbable monofilament, eg, 3-0 Monocryl [poliglecaprone 25]; Ethicon, Somerville, New Jersey, United States). The catheter was then exteriorized through the abdominal wall using the same cannula, and the ligament was secured to the parietal peritoneum with 3 additional fixation sutures (FIGURE 2,FIGURE 3,FIGURE 4,FIGURE 5).

Minilaparotomy technique During the early adoption period, duodenostomy was performed through a 5 − 6 cm upper-abdominal minilaparotomy. In the initial cases, the gastric conduit was elevated before constructing the duodenostomy, which sometimes resulted in a deeper operative field and limited visibility. When necessary, a limited Kocher maneuver was performed to reduce distance and tension between the duodenal bulb and the abdominal wall. The bulb was secured with double purse-strings, and the tube was advanced intraluminally. On the abdominal wall side, the round-ligament sleeve served as a protective tunnel for the catheter and was anchored with interrupted sutures, consistent with the concept used in the laparoscopic approach.

Perioperative management Continuous enteral feeding was initiated on POD 1-2 according to institutional protocol. Patients were monitored for bowel obstruction, infection, or tube dislodgement.

Statistical analysis Continuous variables were assessed for normality with the Shapiro-Wilk test and for homogeneity of variances with the Levene test. Normally distributed data are reported as mean (SD) and were compared with the t test (with the Welch correction when variances were unequal). Non-normally distributed data are reported as median (interquartile range [IQR]) and were compared using the Mann-Whitney test. Categorical variables were compared using the Fisher exact test. Statistical significance was defined as a 2 -sided P value below 0.05. Given the small sample size and low event counts, formal statistical testing was limited to the baseline and early postoperative variables summarized in FIGURE 1; other outcomes were analyzed descriptively. Statistical analyses were performed with JMP 18 Student Edition package (SAS Institute Inc., Cary, North Carolina, United States).

## RESULTS 

Twenty-six patients met eligibility criteria; 15 underwent laparoscopic round-ligament duodenostomy (Lap) and 11 the historical minilaparotomy approach (Mini). All laparoscopic procedures were completed as planned, and catheter placement was technically successful in all cases. Continuous enteral feeding began on POD 1 (median [IQR], 1-2) in the Lap group and POD 2 (median [IQR], 2-6) in the Mini group, with a significantly earlier start in the Lap group ( P = 0.04 ). Given the sample size, the clinical significance of this difference appears limited.

Tube-related infections occurred in 0 of 15 Lap patients and 2 of 11 ( 18.2% ) Mini patients. In the Mini group, exit site erythema with purulent discharge was noted on POD 5 and POD 7, respectively; both cases were managed conservatively with local care and antibiotics, without tube removal or reoperation. No intraperitoneal abscesses or organ/ space infections were identified.

Noninfectious complications (dislodgement, inversion, or obstruction/kinking) occurred in 1 of 15 patients ( 6.7% ) in the Lap group and 1 of 11(9.1%) in the Mini group. Owing to the retrospective design and limited sample size, between--group findings are interpreted descriptively without claims of causality. Short-term outcomes for the between-group comparison are summarized in FIGURE 1.

## DISCUSSION 

In this single-center retrospective exploratory series, a standardized laparoscopic round-ligament duodenostomy synchronized with esophagectomy was feasible and achieved universal technical success in 15 patients with esophageal squamous cell carcinoma. Continuous enteral feeding was initiated early (median [IQR] POD, 1 [1-2]). These exploratory observations are consistent with the hypothesis that avoiding jejunal fixation may help to mitigate fixation--related small bowel obstruction reported with jejunostomy. As a comparative jejunostomy cohort was not included, our conclusions are limited to a descriptive evaluation of duodenostomy outcomes.

Our transition was driven by institutional experience and prior reports describing fixation--related complications after jejunostomy.^3^^,^^4^^,^^5^^,^^6^ Minilaparotomy duodenostomy allowed direct access but provided suboptimal visualization in a deep operative field, and 2 exit-site infections occurred early in our series. In contrast, laparoscopy offers stable magnified views for posterior mediastinal reconstruction and facilitates precise creation of the round-ligament sleeve with secure fixation. These practical factors motivated the transition from minilaparotomy to a laparoscopic approach. The earlier initiation of enteral feeding observed in the Lap cohort (median POD 1 vs POD 2) is consistent with these technical advantages; however, in this small retrospective series without historical controls, this finding should be interpreted cautiously. The absolute difference in timing was modest, and the study was not designed or powered to determine whether an earlier start translated into improvements in clinically meaningful outcomes, such as length of hospital stay, postoperative ileus, or tolerance of feeding advancement. Furthermore, postoperative feeding initiation and escalation were not governed by fully standardized protocols across the study periods, precluding reliable conclusions about the downstream clinical impact of earlier enteral access.

Importantly, tube-related infection occurred in 0 of 15 patients in the Lap group and 2 of 11 individuals (18.2%) in the Mini group (TABLE 1). While causality cannot be inferred, the difference is compatible with the hypothesis that improved visualization and reduced tissue trauma at the exit site may lower the infection risk. 

Early postoperative nutritional support remains central to recovery after esophagectomy.^12^ Although jejunostomy has long been a standard, it carries mechanical risks-including torsion, adhesions, or volvulus at the abdominal wall fixation site-reported in several large series. ^3^^,^^4^^,^^5^^,^^6^ Before introducing duodenostomy, we also used jejunostomy routinely and encountered similar fixation-related issues. Our shift to duodenostomy was intended to minimize fixation-related obstruction, which has been reported to require reoperation in approximately 5.5% of cases and to occur in up to about 12% of patients within 5 years after jejunostomy ^7^^,^^8^^,^^9^; such events may delay recovery, disrupt oncologic therapy, and adversely influence long-term outcomes.^13^^,^^14^^,^^15^ At our institution, jejunostomy had historically been used for enteral access after esophagectomy, but concerns about fixation-related small bowel obstruction and local complications prompted a shift in practice toward duodenostomy. When minimally-invasive esophagectomy was adopted as the standard approach, we standardized round--ligament duodenostomy rather than maintaining parallel jejunostomy protocols. As a result, no contemporary jejunostomy cohort was available for comparison in the present analysis. The primary aim of this exploratory study was therefore not to demonstrate superiority of duodenostomy over jejunostomy, but to describe the technical details of laparoscopic round-ligament duodenostomy, and to explore its short-term safety and feasibility in the context of minimally-invasive esophagectomy. Several alternative strategies have been proposed to reduce the morbidity associated with enteral access after esophagectomy. Gastrostomy with round-ligament coverage has been described as a means of protecting the gastric wall and abdominal entry site, and retrocolic jejunostomy routes have been used to minimize tension and torsion on the jejunal limb. However, these approaches are not always easily integrated into posterior mediastinal reconstruction and still rely on jejunal fixation or gastric wall puncture. In contrast, round-ligament duodenostomy maintains a purely duodenal route and uses the mobilized round ligament as a biologic sleeve and fixation anchor, which may offer a conceptually attractive alternative in the setting of minimally--invasive esophagectomy, albeit on the basis of limited exploratory data. As an alternative, round--ligament duodenostomy avoids jejunal fixation and therefore may reduce these risks.^16^^,^^17^ Early applications via minilaparotomy demonstrated feasibility but raised concerns about visualization and infection in deep fields; accordingly, we performed 11 minilaparotomies beginning in 2021 (2 exit site infections) and adopted a fully laparoscopic approach in 2022.

**TABLE 1:** Patient characteristics and short-term outcomes

Variable		Lap (n = 15)	Mini (*n* = 11)	*P *value
Age, y, mean (SD)		68.3 (9.6)	73.8 (3.8)	0.06
BMI, kg/m²		23.5 (19.4-25.2)	18.6 (17.3-23.6)	0.13
Diabetes mellitus^a ^		5 (33.3)	4 (36.4)	>0.99
Sex	Men	12 (80)	10 (90.9)	0.61
	Women	3 (20)	1 (9.1)	
ASA-PS category	≤ 2	13 (86.7)	10 (90.9)	>0.99
	≥ 3	2 (13.3)	1 (9.1)	
Enteral nutrition initiation, POD		1 (1-2)	2 (2-6)	0.04
Tube-related infection		0	2 (18.2)	0.17
Noninfectious tube-related complications		1 (6.7)	1 (9.1)	>0.99

Data are presented as number (percentage) or median (interquartile range) unless indicated otherwise.

a Diabetes mellitus was defined as a known diagnosis of type 1 or type 2 diabetes treated with diet, oral agents, or insulin.

Abbreviations: ASA-PS, American Society of Anesthesiologists physical status; Lap, laparoscopic round-ligament duodenostomy; Mini, mini-laparotomy duodenostomy; POD, postoperative day

Potential advantages of the laparoscopic round--ligament approach include: 1) no jejunal fixation, potentially reducing fixation-related mechanical complications-relevant when uninterrupted postoperative nutrition is critical ^18^; 2) a lower observed local infection rate in our cohort (0/15 vs 2/11), acknowledging exploratory inference; 3) enhanced visualization and instrument control in the deep operative field; and 4) seamless integration with posterior mediastinal reconstruction without extensive liver manipulation. Our protocol operationalizes these principles via double purse-string fixation at the duodenal bulb, 20 − 30 cm intraluminal advancement, 3-point anchoring on both intestinal and abdominal wall sides using 3-0 absorbable monofilament, routing through a round-ligament sleeve, and confirming the catheter tip by gentle saline injection (and guidewire assistance when needed).^19^^, ^^20^ When the round ligament is short, a short omental bridge can ensure a tension-free course.

This study has several important limitations. First, it was a single-center retrospective analysis with a very small sample size, which substantially limits statistical power. Second, only a small number of variables were investigated, and event counts were too low to permit multivariable modelling; therefore, only univariable analyses were performed. Third, the comparison with the Mini group was historical, introducing potential era and selection biases. In addition, nutritional delivery metrics, patient-reported outcomes, and long-term device data were not available. Moreover, postoperative enteral feeding initiation and escalation were not governed by fully standardized protocols across the study periods, and long-term quality of life or patient--reported outcome data were lacking, which further limits our ability to assess the broader clinical impact of the timing and route of enteral access. Furthermore, we did not have a contemporary jejunostomy comparator cohort, which limits our ability to position round-ligament duodenostomy relative to standard jejunal feeding routes. Taken together, these limitations mean that the present findings should be interpreted as exploratory and hypothesis-generating rather than definitive. Future work should prospectively compare laparoscopic round-ligament duodenostomy with contemporary alternatives (eg, jejunostomy), evaluating time to nutritional targets, device longevity, unplanned interventions, infection, obstruction, cost, and patient-centered outcomes. Confirmation in adequately powered studies is warranted.

## JOURNAL INFORMATION

Videosurgery and Other Miniinvasive Techniques is an official journal of the Videosurgery Foundation.
